# Education shapes the structure of semantic memory and impacts creative thinking

**DOI:** 10.1038/s41539-021-00113-8

**Published:** 2021-12-09

**Authors:** Solange Denervaud, Alexander P. Christensen, Yoed. N. Kenett, Roger E. Beaty

**Affiliations:** 1 Department of Diagnostic and Interventional Radiology, Lausanne University Hospital (CHUV), University of Lausanne (UNIL), Lausanne, Switzerland; 2 Center for Biomedical Imaging (CIBM), Lausanne, Switzerland; 3 Department of Neurology, University of Pennsylvania, Philadelphia, PA USA; 4 Faculty of Industrial Engineering and Management, Technion—Israel Institute of Technology, Haifa, Israel; 5 Department of Psychology, Pennsylvania State University, Philadelphia, PA USA

**Keywords:** Human behaviour, Long-term memory

## Abstract

Education is central to the acquisition of knowledge, such as when children learn new concepts. It is unknown, however, whether educational differences impact not only what concepts children learn, but how those concepts come to be represented in semantic memory—a system that supports higher cognitive functions, such as creative thinking. Here we leverage computational network science tools to study hidden knowledge structures of 67 Swiss schoolchildren from two distinct educational backgrounds—Montessori and traditional, matched on socioeconomic factors and nonverbal intelligence—to examine how educational experience shape semantic memory and creative thinking. We find that children experiencing Montessori education show a more flexible semantic network structure (high connectivity/short paths between concepts, less modularity) alongside higher scores on creative thinking tests. The findings indicate that education impacts how children represent concepts in semantic memory and suggest that different educational experiences can affect higher cognitive functions, including creative thinking.

## Introduction

Early experience is of paramount importance for later cognitive and emotional outcomes^1^. In this period of high brain plasticity^2,3^, children’s knowledge is acquired efficiently through statistical learning^4,5^ and it is significantly shaped by interactions with the environment^6^. Despite the importance of experience on semantic knowledge (i.e.,^7^), few researches have focused on the role of school education in influencing not only how children *acquire* new knowledge, but also how they come to *represent* knowledge in long-term (semantic) memory. The organization of semantic memory plays a key role in higher cognitive functions, such as creative thinking^8^. In the present research, we apply network science methods to investigate how different educational approaches, namely traditional and Montessori approaches, shape 5–12-year-old children’s internal knowledge representation in semantic memory (i.e., concept learning) and their ability to think flexibly and creatively.

Montessori and traditional education can both be of high quality, but their approaches differ with respect to *concept learning*—an important feature of cognitive development supporting the acquisition of new vocabulary and crystallized knowledge. Montessori education focuses on self-directed and uninterrupted learning activities that children perform within multi-age classes^9,10^. Children in these classes routinely engage in interdisciplinary, discovery-based work to learn new concepts (e.g., draw the outline of the continents, write their names, and classify them according to their population size), such as conducting experiments in and out of the class, often with minimal (but guiding) feedback from teachers. According to the Swiss educational plan, traditional education focuses on teacher-directed learning activities, introducing successively different topics (e.g., language, writing, geography, math) that children perform within single-age classes. Children in these classes are asked to learn and memorize concepts (i.e., rote learning), knowledge on which they are regularly tested and evaluated with grades (starting from 6 years of age). When comparing Montessori and traditional educational approaches, Montessori classes have been shown to promote improved academic outcomes, socio-emotional learning, and divergent and/or convergent creativity^11–15^. Such effects raise questions about how educational experiences shape children’s fundamental cognitive processes, such as concept learning.

Environmental interaction plays an essential role in how children first learn about words and concepts^7^. For example, parents’ socioeconomic level is tightly related to preschoolers’ vocabulary level^16^, primarily until around the age of six^17^. As the child develops, the experience within the environment assumes an important part in the continuation of concept learning^18^. Indeed, semantic memory shows a 1.6 fold increase across elementary school years, with ~3200 root words being acquired between the second and the fifth grades^19^. During this period, the child learns not only the root word and its meaning, but how these words relate to other words, establishing deep knowledge representation^20^. During this crucial period of 6 to 12 years, the child encodes on average 800–900 new concepts a year, representing the building blocks of knowledge that form an interconnected structure in semantic memory^21^. Individual differences in semantic representation are known to impact many important cognitive abilities (e.g., creative thinking) by influencing how knowledge is retrieved from memory^22^.

In recent years, a new field of cognitive network science has prompted a paradigm shift in the study of higher cognitive processes^23,24^. Cognitive network methods can be used to estimate the organization of concepts in semantic memory. For example, using a simple verbal fluency task, where participants name as many words as they can for one given category in 1 min (e.g., foods), it is now possible to estimate an individual’s knowledge representation using computational network science tools^25^. Semantic network analyses depict each concept (name) as a node, and relations between them as edges. Thus, the less related the concepts, the longer the edges (e.g., pear and avocado vs. pear and apple), but also the slower the participant will be to report their relationship^24^.

Network science measures can provide insight about differences in semantic network structure. For example, network measures can quantify whether concepts tend to be more isolated or closely related to others (clustering coefficient; CC), whether information flow is more efficient (average shortest path length; ASPL), or whether concepts’ integration is rigid vs. flexible modularity; Q;^24^. Beyond simply representing semantic memory, network science tools allow researchers to study the psychological and behavioral consequences of different memory structures. For example, adults with a more efficient semantic network structure—characterized by high connectivity (higher CC, lower Q) and short path lengths between concepts (lower ASPL)—show higher levels of the personality trait openness to experience, i.e., the tendency to seek out and enjoy novel experiences^25^.

Moreover, it has been robustly demonstrated that higher creative individuals (assessed via self-reported achievement and cognitive measures) also show this flexible network structure, which is likely conducive to creative thinking due to “closer” representation of otherwise “distant” concepts in semantic memory^8^. In contrast, less creative individuals show a more rigid memory structure, marked in part by high modularity of concepts into canonical subcategories in memory (e.g., animals). Together, these data suggest that a more flexible knowledge representation is associated with important outcomes, with likely implications for cognition and behavior across the lifespan.

How do concepts come to be represented differently in semantic memory? And what is the initial source of individual differences in semantic memory structure? In the present research, we examine one potential but underexplored source of variation: education. As a test case, we focus on children from Montessori classes and compare them to children from traditional classes. To account for a possible selection-bias of private Montessori classes with high pedagogical standards, public traditional classes were selected among high socioeconomic city areas and selected for their high-quality teaching practices (teachers trained in renowned pedagogical centers). Although typically developing children—from similar socioeconomic backgrounds—may not show notable differences on tests of short-term retention and cognitive ability, their dynamic knowledge representation may show incremental change over time^26^. Because children from Montessori classes learn through self-directed engagement in naturalistic and interdisciplinary activities, we hypothesize that semantic concept integration would diverge from the more traditional, adult-directed curriculum, in being more enriched (higher CC), interconnected (shorter ASPL), and flexible (lower Q).

Given the long-term behavioral and cognitive implications associated with memory structure, and based on previous findings in adults^27^, we specifically addressed the question of whether variation in semantic memory structure tracks cross-sectional improvements in other cognitive abilities over time, with a focus on creative thinking. According to previous work^11,28^, we expect children from Montessori classes to present higher convergent and divergent creative skills than children from traditional classes. For the first time, we therefore examine whether educational differences shape knowledge representation and corresponding creative abilities.

## Results

### Participants

We began by testing for potential group differences between children from Montessori and traditional classes. Across gender, age, nonverbal intelligence, and parental SES, no significant differences were found, revealing comparable groups (Table 1).

**Table 1:** Demographic and nonverbal intelligence data for the Montessori—(M) and traditionally—(T) schooled children.

	Group		*X*^2^ or *t* test	*p* values	Cohen’s d
	M	T			
N (girls)	36 (16)	31 (16)	0.34	0.58	
Age [years]	9.1 (2.3)	8.6 (2.0)	1.03	0.31	0.25
min, max	5.5–14.6	5.2–12.3			
Nonverbal Intelligence [score]	33.2 (3.45)	32.2 (3.59)	2.28	0.30	0.81
SES [score]	7.03 (1.41)	6.83 (1.46)	0.54	0.59	0.14

Mean and SD in parentheses.

### Verbal fluency

Having established that the two groups were demographically and intellectually similar, we next assessed their performance on the verbal fluency task (animal category; Table 2). While children experiencing Montessori education produced a similar number of responses (M = 16.4, SD = 5.0) than their peers experiencing a more traditional education (M = 14.8, SD = 5.8), they gave more unique responses (105) than their peers from traditional classes (87; *p* = 0.023). Of these unique responses, children experiencing Montessori education provided more unique responses that the children experiencing traditional education did *not* provide (37) than vice versa (19).

**Table 2:** Verbal fluency and creativity data for the Montessori—(M) and traditionally—(T) schooled children.

	Group		*t* test or *X*^2^	*p* values	Cohen’s d or Phi
	M	T			
Mean total number of responses	16.4 (5.0)	14.8 (5.8)	1.23	0.225	0.30
Number of unique responses	37/105	19/87	5.16	0.023	0.28
Creativity divergent	9.6 (3.7)	7.1 (5.1)	2.34	0.02	0.58
Creativity convergent	5.3 (1.7)	2.6 (1.3)	7.10	<0.001	1.74

Mean and SD in parentheses.

### Creativity assessment

Next, we tested whether education influenced creative thinking (Table 2). We found that the groups differed on both divergent and convergent creativity tests, with the Montessori sample scoring on average higher (M_div_ = 9.6, M_conv_ = 5.3) than the traditional sample (M_div_ = 7.1, M_conv_ = 2.6), *t* (64) = 3.24, *p* = 0.02, *d* = 0.58, and *t* (64) = 7.10, *p* < 0.001, *d* = 1.74, respectively (Table 2). Divergent and convergent creativity scores were positively correlated with the total number of responses on the verbal fluency test, *r* = 0.27, *p* = 0.03, and *r* = 0.36, *p* = 0.003, respectively, replicating past work^29^.

### Network analysis

**Fig. 1:**
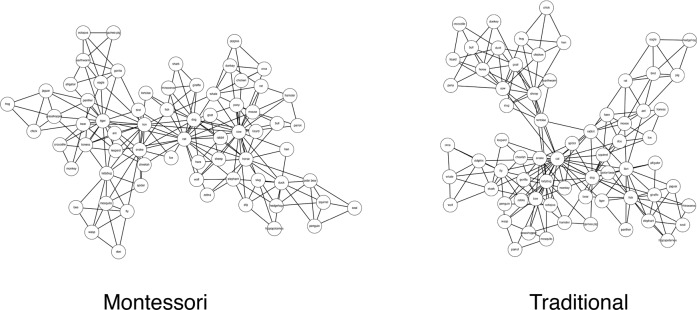
Semantic Network. 2D Visualization of the semantic networks of the Montessori and traditional groups.

Finally, we estimated the semantic networks of the Montessori and traditional groups (Fig. 1). The networks were visualized (Fig. 1) using the Cytoscape software^30^. In these 2D visualizations, nodes are represented by the respective circles and edges between them are represented by lines. Since these networks are undirected and weighted, the edges convey symmetrical (i.e., bidirectional) similarities between two nodes. A qualitative inspection of these network visualizations illustrates that the semantic network of the Montessori group is more condensed (nodes are closer together) and less modular (the network has fewer subcomponents) than the semantic network of the traditional group.

To statistically validate our results, we incorporated two complimentary statistical significance testing methods. First, we compared the measures of our empirical semantic networks to those of randomly generated networks. Across all three networks, the simulated random network analysis revealed that the empirical network measures (ASPL, CC, and Q) for both groups were statistically different from random networks (all *p*’s < 0.001).

**Fig. 2:**
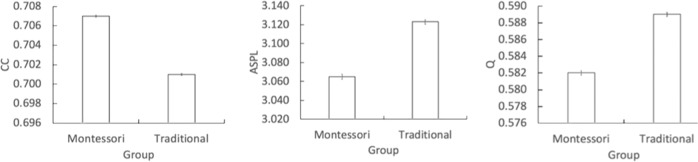
Case-wise bootstrapping analysis for the Montessori and traditional groups. Clustering coefficient (CC, left), average shortest path length (ASPL, center), and modularity (Q, right). *X*-axis: school group; *Y*-axis: dependent variables (CC, ASPL, and Q; error bars denote standard error). The range of scores on the different *Y*-axes are measure-specific and should not be compared across measures. Furthermore, the *Y*-axes across the three measures do not start at zero as to clarify the difference in measures across the two groups.

Second, case-wise bootstrap network analyses evaluated the differences in the network measures between both groups (Fig. 2). An independent-groups t test revealed that the Montessori-schooled children had a significantly higher CC (M = 0.707, SD = 0.01) than the traditional-schooled children (M = 0.701, SD = 0.01), *t* (1998) = 11.33, *p* < 0.001, *d* = 0.51. Additionally, the Montessori-schooled children had a significantly lower ASPL (M = 3.07, SD = 0.26) than the traditional-schooled children (M = 3.12, SD = 23), *t* (1998) = −5.32, *p* < 0.001, *d* = 0.24. Finally, the Montessori-schooled children had a significantly lower Q (M = 0.582, SD = 0.03) than the traditional-schooled children (M = 0.589, SD = 0.02), *t* (1998) = −7.07, *p* < 0.001, *d* = 0.32. Thus, compared to the traditional group, the semantic network of the Montessori group was more interconnected (higher CC), with shorter paths between concepts (lower ASPL) and fewer subcommunities (lower Q).

## Discussion

Despite the central importance of education for child development, whether educational differences impact how children represent knowledge has remained unknown. The present research provides the first evidence to indicate that education shapes both the structure of children’s semantic memory and their ability to think creatively. As a test case, we focused on children experiencing a common child-centered-constructivist education (i.e., Montessori) versus traditional education. While both can be of high quality, their approach to concepts learning differ in many aspects. We found that compared to children experiencing a traditional education, children experiencing Montessori education showed a more “flexible” semantic network structure, characterized by higher connectivity and shorter paths between concepts, as well as lower modularity—a network structure that is conducive to connecting remotely-associated concepts when thinking creatively^8^. Consistent with this view, the Montessori class showed higher scores on tests of both divergent and convergent creative thinking. Critically, the groups were matched on nonverbal intelligence and demographic/socioeconomic factors, indicating that differences in semantic network and creative thinking are specific to school education and not due to extraneous group differences. The findings suggest that educational practices have an impact on the structure of semantic memory, with implications for higher cognitive functions such as creativity.

The findings may reflect differences in how knowledge is taught at school. In a more interdisciplinary and active way of learning, concepts may be perceived as more dynamic and connected, offering children a more flexible, broader understanding and interpretation of concepts—beyond what they may learn in a more directed education (leading to more passive learning) that focuses on distinct disciplines successively. Differences in knowledge representation may also be related to other factors, such as free movement and active learning, found in Montessori education, or the uninterrupted work (providing no time limit or stress to embody concepts), peer-peer teaching (rehearsal of concepts, impact on attentional processes), and multi-age classes (higher diversity in language heard). Together, each of these educational features may gradually come to shape and/or train the structure of children’s semantic networks, with continued expansion and integration of networks with learning over time^31,32^. Future work is critical to better decipher which factors provide children the opportunity to grow enriched knowledge representation, and why.

With respect to creativity, we found that education differences played a significant role in affecting children’s creative thinking. Across both divergent and convergent creativity tests, children from Montessori classes showed higher scores compared to their peers from affluent traditional classes, corroborating previous studies on creativity in Montessori students^11,12^. Similar to semantic abilities, specific teaching practices found in Montessori classes may foster higher creativity skills, such as the error-and-trial approach (children need to solve problems by and for themselves), the peer-peer tutoring in multi-age classes (higher social diversity, different points of view), the multisensory didactic material (using more than two senses to learn and later create), the absence of time pressure (uninterrupted three working hours), and the project-based learning (self-directed exploration). Which of these factors explain higher creative thinking is not yet known. However, we suspect that it may even be the combination of these, and may be even other/unknown factors, that allow children to access and train their creative abilities. Future work is crucially needed in that direction.

For now, the semantic network and creativity results are consistent with studies in adults, which have found that high creative ability is characterized by the same flexible network structure observed in Montessori students from the current study, i.e., high connectivity and short paths between concepts, with less modularity of concepts into discrete subcommunities^8,33^. This flexible network structure is thought to be conducive to creative thinking, allowing concepts to be more efficiently accessed and flexibly combined, compared to a network structure that is more spread out and modular^8^. An important direction for future research will be to directly examine the interactions between education, semantic structure, and creative thinking. Importantly, given the group-based approach to modeling semantic networks, the current study could not link individual network metrics to creativity scores. Future research should conduct longitudinal analyses to examine how semantic network structure relates to the development of creative thinking over time in different educational contexts, and to expand this investigation to other higher-level cognitive abilities (e.g., language skills in general).

Although we could not directly link semantic network metrics with creativity scores, we found that children from Montessori classes, despite an average number of responses similar to their peers from traditional classes (which all correlated with creativity scores), produced more unique verbal fluency responses, potentially reflecting a greater depth and breadth of semantic knowledge (at least within the animal category). While Montessori education is not known for promoting learning about animals more than Swiss traditional classes (i.e., specific material exposed in the environment), this finding corroborates previous studies reporting the early acquisition of reading skills in Montessori schoolchildren, and later higher outcomes in language tasks^12–14,34^. Montessori education offers a language curricula that starts as early as the children gain curiosity for the sounds and letters, around 3.5 years of age^9,10^. Children in these classrooms are given educational materials to learn real-life concepts by associating names with objects (e.g., specific leaf shape with “obovate”). Over time, the children learn to categorize concepts (e.g., plant phylogeny), while conducting experiments in and out of the classroom (e.g., seeding plants, collecting leaves). It may be that while children’s abstraction and semantic memory grow and mature, a form of experience-dependent plasticity comes to deepen their understanding of concepts, dynamically shaping semantic networks as new concepts are learned and connections are made between them. Enriched language curricula—with self-directed and naturalistic activities that introduce successive levels of concept knowledge—may help children optimally train and expand the breadth and depth of their semantic knowledge (perception, conceptualization, generalization). The structure of semantic networks may also benefit from the diversity of social interactions found in multi-age classes (such as Montessori), where fostered peer-to-peer teaching may hasten the assimilation of vocabulary^35^. Future research should identify the relative contributions of classroom curricula and social factors in influencing the development of semantic networks and related cognitive abilities, such as creativity.

The present study has some caveats. First, our study relies on a cross-sectional design. While these results constitute early evidence that semantic networks are highly permeable to educational experience, longitudinal studies are needed. Second, our study included only one semantic category (animals). However, it would be of interest to study other semantic domains, based on child-centered interests (i.e., leisure activities, hobbies) to explore “out-of-class” knowledge representation, and to assess any transfer effects from academic knowledge representation to self-directed learning of new concepts (i.e., do children learn a pattern of semantic organization, or do they learn a model of ideas independently from each other). Finally, we cannot infer which features from the Montessori education help children to build a flexible and extensive semantic network. Future research could investigate specific educational features, such as early training in reading, learning concepts from active and naturalistic activities, and peer-to-peer learning. These studies can shed light on how educators can foster “flexible” knowledge representation within schools, creating learning environments that promote deep learning, and creativity.

## Method

### Participants

A total of 67 children participated in the current study (M_*age*_ = 9.31, SD = 2.23, 47.8% girls) through the University Hospital of Lausanne research pool as part of a broader research project on education and neurocognitive development. Children were compensated with a ~30 USD gift voucher for completion of the study. Inclusion criteria were schooling system (participants had to be enrolled in Montessori or in traditional classes from the early years on, in the case of the youngest children, or for at least 3 years), age (5–14 years of age); exclusion criteria were parental report of learning disabilities or sensory impairment. To account for variability in our measures due to nonverbal intelligence, or socioeconomic background, we controlled for between-group homogeneity in nonverbal intelligence (black and white short version of the Progressive Matrices^36^) and family socioeconomic status (both parents’ education levels (score from 1 to 5) and current job (score from 1 to 4); scores were summed and averaged between both parents (max 9), with higher scores denoting higher SES).

A chi-square test of goodness-of-fit was performed to determine whether the gender ratios were equal between the Montessori and traditional group of children. In addition, we conducted *t* tests (independent or Welch’s, according to the preliminary data check with Q–Q plots and Levene’s test) with a 95% confidence interval (CI) for the mean difference to test for significant differences between the groups on demographic variables and nonverbal intelligence.

## Materials

### Verbal fluency

Children completed verbal fluency task. Category verbal fluency tasks have been widely used to efficiently assess semantic network organization^37^. Consistent with traditional task administration^38^, each child had 60 s to name as many animals as he/she could. Based on previous work in children, we targeted the animal category^39^. Children spoke their responses out loud, which were recorded (and later transcribed) by an experimenter. For each child, fluency data was preprocessed using the *SemNA* pipeline in R^37^. Repetitions or variation on roots were converged and non-category members were excluded from the final analysis. Number of responses per participant were summed (total number of responses). An independent *t* test was used to determine whether both groups differ on average number of responses and number of unique responses, permitting a test of whether education affects the types of words that children retrieve when searching semantic memory.

### Creativity assessment

To assess creative thinking, children completed divergent and convergent creativity tasks from the Evaluation of Potential Creativity^40^. Divergent thinking reflects the ability to think of ideas that differ from one another; convergent thinking reflects the ability to think of a single creative solution. Performance on such creative thinking tasks has been shown to predict both academic^41^ and creative^42^ achievement. In the divergent thinking task, the child was asked to draw as many different drawings as possible from one imposed abstract form (i.e., incomplete shape), within 10 min. The final score was the sum of all valid drawings. In the convergent thinking task, the child had to select three different abstract forms out of eight to create an original drawing that combined them, within 15 min. Three blind judges scored the drawings for originality following the EPoC scoring manual (inter-rater agreement; Krippendorff’s alpha = 0.905). Independent *t* tests were computed on each creativity score (divergent and convergent) to test for between-group differences. Pearson’s correlations were computed between each creativity score and the verbal fluency metrics to test whether creative thinking relates to the quantity and quality of words retrieved from semantic memory.

### Network analysis

The semantic verbal fluency data of both groups were analyzed using a semantic network approach^43,44^. In this approach, each node represents a category exemplar (e.g., frog) and edges represent associations between two exemplars. These associations are the tendency of the sample to generate exemplar *b* (e.g., toad) when they have also generated exemplar *a* (e.g., frog). All network analyses were conducted in R using a publicly-available pipeline to analyze semantic fluency data as networks^37^, with the following steps:

#### Semantic network estimation

The processed data were transferred into a binary response matrix, where columns represent the unique exemplars given by the sample and rows represent participants; the response matrix is filled out by 1 (if an exemplar was generated by that participant) and 0 (if that exemplar was not generated). To control for confounding factors (such as different nodes or edges in both groups), as in previous studies^25,43^, the binary response matrices only include responses that are given by at least two participants in each group. Then, to avoid the two groups including a different number of nodes, which may bias comparison of network parameters, responses in the binary matrices were equated, so that the networks of both groups in each sample are compared using the same nodes^37^.

Next, we computed a word association matrix for each group using the cosine similarity. The cosine similarity is commonly used in related to Pearson’s correlation, which can be considered as the cosine between two normalized vectors. With the cosine similarity measure, all values are positive ranging from 0 (two responses do not co-occur) to 1 (two responses always co-occur). For both groups, each element in the word association matrix, *A*_*ij*_, represents the cosine similarity or the co-occurrence between response *i* and *j*.

Finally, using these word association matrices, we applied the triangulated maximally filtered graph TMFG^37^; to minimize noise and potential spurious associations. The TMFG method filters the word association matrices to capture only the most relevant information (i.e., removal of spurious associations and retaining the largest associations) within the original network. This approach retains the same number of edges between groups (i.e., 3*n*–6, where *n* equals the number of responses), which avoids the confound of difference network structures being due to a different number of edges^43^. This resulted in a 68 nodes network with 198 edges for both groups.

#### Semantic network analyses

The SemNA pipeline in R^37^ was used to compute the CC, ASPL, and Q measures for both groups. *Clustering Coefficient* (CC) refers to the extent that neighbors of a node will themselves be neighbors (i.e., a neighbor is a node *i* that is connected through an edge to node *j*). Higher clustering coefficient indicates a more interconnected semantic network^24^. *Average Shortest Path Length* (ASPL) refers to the average shortest number of steps (i.e., edges) needed to traverse between any pair of nodes; the higher the ASPL, the more spread out a network is. Previous research has shown that the ASPL in semantic networks corresponds to participants’ judgments as to whether two concepts are related to each other^24^. *Modularity* (Q) estimates how a network breaks apart (or partitions) into smaller sub-networks or communities^24^. Q measures the extent to which the network has dense connections between nodes within a community and sparse (or few) connections between nodes in different communities. Thus, the higher Q, the more the network breaks apart to subcommunities. Such subcommunities can be thought of as subcategories in a semantic network (e.g., farm animals in the “animals” category). Previous research has shown that modularity in semantic networks is inversely related to a network’s flexibility^8^.

#### Statistical analysis

We applied two complementary approaches for comparing the networks: comparisons against random networks and bootstrap network comparisons. Our first approach, comparisons against random networks, is used to determine whether the network measures (ASPL, CC, and Q) observed in the groups’ networks are different from what would be expected from a random network with the same number of nodes and edges^45^. This approach iteratively simulates Erdös–Rényi random networks (e.g., 1000 networks) with the same number of nodes and edges with a fixed edge probability^46^. For each simulated random network, network measures (i.e., CC, ASPL, and Q) are computed, resulting in a sampling distribution of these measures for the random network. The empirical group network measures are then compared against the respective distributions of the random network measures. The Erdös–Rényi random network model does not make any assumptions regarding the structure of the network, which makes it a useful null model to test against^46^. That is, it tests whether the network structure for a specific network measure could be generated from a random network model.

Second, for each dataset, we used a bootstrapping approach to simulate and compare semantic networks of the two groups^25,43^. We applied the case-wise bootstrap method, which resamples from the original sample *with replacement*. Each bootstrap sample has as many participants as the original sample but with some participants being included more than once and others not being included at all. Networks are estimated on each bootstrap sample following the network construction approach described above. Network measures are then computed for these networks. This process repeated iteratively for 1000 times.

Similar to the comparisons against random networks, these bootstrapped networks form a sampling distribution of the network measures for both groups, but solely based on the empirical data. Independent-group *t* tests are then applied to statistically examine the difference between the distribution of the three network measures (CC, ASPL, and Q) across both groups. Cohen’s *d* effect sizes are reported following^47^: small = 0.20, moderate = 0.50, and large = 0.80.

### Procedure

This study was approved by the ethical committee of the CER-Vaud (Switzerland). Written informed consent to take part in the study was obtained from parents and oral consent from children, who acknowledged that they were free to withdraw at any time without penalty. Group variables (e.g., SES) were collected through online questionnaires completed at home after the experiment. Verbal fluency and creativity data were collected by the experimenter in school, in a dedicated room, with the child seated at a table next to the experimenter. Each child was randomly assigned to complete one of the two tasks first.

### Reporting summary

Further information on research design is available in the Nature Research Reporting Summary linked to this article.

## Supplementary information

Reporting Summary

## Data Availability

Data and materials for this study have not been made publicly available, but can be shared upon request. The design and analysis plans were not preregistered.
