# Supplementary material for: Education shapes the structure of semantic memory and impacts creative thinking
Source: NPJ Sci Learn. 2021 Dec 9;6:35. doi: 10.1038/s41539-021-00113-8 (PMC8660875; doi:10.1038/s41539-021-00113-8)
Supplement: Supplementary file 1 — Reporting Summary [file 41539_2021_113_MOESM1_ESM.pdf]

nature portfolio

Corresponding author(s): Solange Denervaud

Last updated by author(s): Aug 4, 2020

Reporting Summary

Nature Portfolio wishes to improve the reproducibility of the work that we publish. This form provides structure for consistency and transparency in reporting. For further information on Nature Portfolio policies, see our Editorial Policies and the Editorial Policy Checklist.

Statistics

For all statistical analyses, confirm that the following items are present in the figure legend, table legend, main text, or Methods section.

n/a	Confirmed	
☐	☒	The exact sample size (*n*) for each experimental group/condition, given as a discrete number and unit of measurement
☐	☒	A statement on whether measurements were taken from distinct samples or whether the same sample was measured repeatedly
☒	☐	The statistical test(s) used AND whether they are one- or two-sided<br>*Only common tests should be described solely by name; describe more complex techniques in the Methods section.*
☐	☒	A description of all covariates tested
☐	☒	A description of any assumptions or corrections, such as tests of normality and adjustment for multiple comparisons
☐	☒	A full description of the statistical parameters including central tendency (e.g. means) or other basic estimates (e.g. regression coefficient) AND variation (e.g. standard deviation) or associated estimates of uncertainty (e.g. confidence intervals)
☐	☒	For null hypothesis testing, the test statistic (e.g. *F*, *t*, *r*) with confidence intervals, effect sizes, degrees of freedom and *P* value noted<br>*Give P values as exact values whenever suitable.*
☒	☐	For Bayesian analysis, information on the choice of priors and Markov chain Monte Carlo settings
☐	☒	For hierarchical and complex designs, identification of the appropriate level for tests and full reporting of outcomes
☐	☒	Estimates of effect sizes (e.g. Cohen's *d*, Pearson's *r*), indicating how they were calculated

Our web collection on statistics for biologists contains articles on many of the points above.

Software and code

Policy information about availability of computer code

Data collection	Children completed verbal fluency task, creativity task, and fluid intelligence task.<br>Parental report for SES
Data analysis	For each child, fluency data was preprocessed using the SemNA pipeline in R, then semantic verbal fluency data of both groups were analyzed using a semantic network approach. Creativity was scored and compared between groups. Group homogeneity for fluid intelligence and SES was tested.

For manuscripts utilizing custom algorithms or software that are central to the research but not yet described in published literature, software must be made available to editors and reviewers. We strongly encourage code deposition in a community repository (e.g. GitHub). See the Nature Portfolio guidelines for submitting code & software for further information.

Data

Policy information about availability of data

All manuscripts must include a data availability statement. This statement should provide the following information, where applicable:

- Accession codes, unique identifiers, or web links for publicly available datasets
- A description of any restrictions on data availability
- For clinical datasets or third party data, please ensure that the statement adheres to our policy

Data and materials for this study have not been made publicly available, but can be shared upon request. The design and analysis plans were not preregistered.

Field-specific reporting

Please select the one below that is the best fit for your research. If you are not sure, read the appropriate sections before making your selection.

☐ Life sciences ☒ Behavioural & social sciences ☐ Ecological, evolutionary & environmental sciences

For a reference copy of the document with all sections, see nature.com/documents/nr-reporting-summary-flat.pdf

Behavioural & social sciences study design

All studies must disclose on these points even when the disclosure is negative.

Study description	Here we leverage computational network science tools to study hidden knowledge structures of 67 children from two distinct educational backgrounds— Montessori and traditional, matched on socioeconomic factors and non-verbal intelligence— to examine how educational experience shape semantic memory and creative thinking.
Research sample	A total of 67 children participated in the current study (Mage = 9.31, SD = 2.23, 47.8 % girls)
Sampling strategy	There is not much work on children's semantic network, and only one study (to our knowledge) in schoolchildren; "Revealing the Role of Divergent Thinking and Fluid Intelligence in Children's Semantic Memory Organization" from Rastelli, Greco, & Finocchiaro, 2020. Our sample-size is similar to this recent work of comparable mean age (around 60 children, split into two groups).
Data collection	Category verbal fluency tasks have been widely used to efficiently assess semantic network organization35. Consistent with traditional task administration, each child had 60 seconds to name as many animals as he/she could. Based on previous work in children, we targeted the animal category. Children spoke their responses out loud, which were recorded (and later transcribed) by an experimenter. To assess creative thinking, children completed divergent and convergent creativity tasks from the Evaluation of Potential Creativity. Divergent thinking reflects the ability to think of ideas that differ from one another; convergent thinking reflects the ability to think of a single creative solution.
Timing	start: november 2018<br>end: september 2019
Data exclusions	No data excluded
Non-participation	exclusion criteria were parental report of learning disabilities or sensory impairment.
Randomization	NA

Reporting for specific materials, systems and methods

We require information from authors about some types of materials, experimental systems and methods used in many studies. Here, indicate whether each material, system or method listed is relevant to your study. If you are not sure if a list item applies to your research, read the appropriate section before selecting a response.

Materials & experimental systems

n/a	Involved in the study
☒	☐ Antibodies
☒	☐ Eukaryotic cell lines
☒	☐ Palaeontology and archaeology
☒	☐ Animals and other organisms
☐	☒ Human research participants
☒	☐ Clinical data
☒	☐ Dual use research of concern

Methods

n/a	Involved in the study
☒	☐ ChIP-seq
☒	☐ Flow cytometry
☒	☐ MRI-based neuroimaging

Human research participants

Policy information about studies involving human research participants

Population characteristics	Inclusion criteria were schooling system (participants had to be enrolled in Montessori or in traditional classes from the early years on, in the case of the youngest children, or for at least 3 years), age (5 to 14 years of age)
Recruitment	through the University Hospital of Lausanne research pool as part of a broader research project on education and neurocognitive development
Ethics oversight	This study was approved by the ethical committee of the CER-Vaud (Switzerland).

Note that full information on the approval of the study protocol must also be provided in the manuscript.
